# Management of patients with low back pain: a survey of French chiropractors

**DOI:** 10.1186/2045-709X-22-13

**Published:** 2014-03-28

**Authors:** Michel Debarle, Rémi Aigron, Laure Depernet, Amandine Guillemard, Thomas Véron, Charlotte Leboeuf-Yde

**Affiliations:** 1 Institut Franco-Européen de Chiropraxie, 24 blv Paul Vaillant Couturier, F-94200 Ivry sur Seine, France

**Keywords:** Survey, Chiropractors, Consensus, Low back pain

## Abstract

**Background:**

Little is known about the level of consensus within the French chiropractic profession regarding management of clinical issues. A previous Swedish study showed that chiropractors agreed relatively well on the management strategy for nine low back pain scenarios. We wished to investigate whether those findings could be reproduced among French chiropractors.

**Objectives:**

1. To assess the level of consensus among French chiropractors regarding management strategies for nine different scenarios of low back pain. 2. To assess whether the management choices of the French chiropractors appeared reasonable for the low back pain scenarios. 3. To compare French management patterns with those described in the previous survey of Swedish chiropractors.

**Method:**

A postal questionnaire was sent to a randomly selected sample of 167 French chiropractors in 2009. The questionnaire described a 40-year old man with low back pain, and presented nine hypothetical short-term outcome scenarios and six possible management strategies. For each of the nine scenarios, participants were asked to choose the management strategy that they would recommend. The percentages of respondents choosing the different management strategies were identified for each scenario. Appropriateness of the chosen management strategy was assessed using predetermined “best practice” for each scenario. Consensus was arbitrarily defined as “moderate” when 50- 69% of respondents agreed on the same management choice for a scenario, and “excellent” when 70% or more provided the same answer.

**Results:**

Excellent consensus was achieved for only one scenario, and moderate consensus for two scenarios. For five of the nine scenarios, the most common answers were in agreement with the “best practice” management strategies. Consensus between the French and Swedish responses on the most appropriate management was seen in five of the nine scenarios and these were all in agreement with the expected answer.

**Conclusion:**

There was reasonable consensus among the French chiropractors in their choice of treatment strategy for low back pain and choices were generally in line with “best practice”. The differences in response between the French and Swedish chiropractors suggest that cultural and/or educational differences influence the conceptual framework within which chiropractors practice.

## Abstract (fr)

**Resume:**

**Contexte**

Peu d’informations du niveau de consensus de prise en charge des chiropracteurs français sont connues. Une étude suédoise a montré un consensus de stratégie de prise en charge (SPC) de chiropracteurs pour neuf scenarii de douleur de bas du dos. Ces résultats pouvaient-ils être reproduits avec des chiropracteurs français?

**Objectifs**

1) Mesurer le taux de consensus de SPC pour neuf scenarii de douleur de bas du dos. 2) Evaluer comment le choix de la SPC des chiropracteurs français apparaît raisonnable pour les neuf scenarii de douleur de bas du dos. 3) Comparer la SPC des chiropracteurs français à celle décrite dans l’étude suédoise.

**Méthode**

En 2009, un questionnaire, envoyé à une sélection randomisée de 167 chiropracteurs français, décrivait le cas un homme de 40 ans présentant une douleur de bas du dos, neuf scenarii hypothétiques d’évolution de la douleur et six SPC possibles. Pour chacun des scenarii, les participants choisissaient la SPC qui leur semblait appropriée. Le pourcentage pour chaque choix de SPC a été calculé. La pertinence du choix de SPC a été évaluée par rapport à des stratégies prédéterminées selon des critères de « bonne pratique » pour chaque scenario. Le consensus a été arbitrairement choisi comme « modéré » pour 50 à 69% de réponses concordantes, et « excellent » pour 70% et plus.

Un excellent consensus n’a été trouvé que pour un seul scenario et un consensus modéré pour deux scenarii. Pour cinq des neuf scenarii, la réponse la plus fréquente était en accord avec le modèle de « bonne pratique ». Le consensus entre les chiropracteurs français et suédois pour la SPC la plus appropriée a été vu pour cinq des neuf scenarii, ce qui est conforme au résultat attendu.

Il y a eu un consensus raisonnable des chiropracteurs français dans leur choix de SPC. Et leur choix de SPC était en agrément avec notre définition de «bonne pratique ». La différence entre les réponses des chiropracteurs français et suédois suggère des différences culturelles et/ou éducatives influençant la trame conceptuelle d’exercice de la chiropraxie.

## Background

Although chiropractic concepts were traditionally anchored in the North American culture, several institutions in other parts of the world now train their own chiropractors. As a consequence, new groups of chiropractors have emerged with little or only indirect professional influence from the North American chiropractic colleges.

When chiropractors are trained in non-English speaking countries, it is possible that the traditional English-language chiropractic literature is only partially understood or incompletely covered. Add to this any culturally driven concepts that affect attitudes and behaviour regarding health and disease, and the scenario is set for potentially very different chiropractic approaches. It is therefore relevant to investigate whether the modern European chiropractic profession is a homogeneous group, despite in some cases having severed the bands with its “alma mater”.

The French chiropractic profession has had its own chiropractic college since the early 1980s. This college is accredited by the European Council on Chiropractic Education, and as such it fulfils certain standards that are common to chiropractic institutions all over the world.

The English language is not highly prioritized in French schools, however, and the reading of English literature is difficult for most French students and even for their lecturers and tutors. The typical North American chiropractic information sources on chiropractic concepts would thus tend not to be used to any great extent and it may be difficult to follow scientific developments in the English-language literature. Furthermore, until recently the practice of chiropractic was illegal in France and the chiropractic profession is small and quite isolated, even marginalized, from other health care professions. It would thus not be surprising if French chiropractors differed from chiropractors trained in English-speaking institutions.

Little is known about clinical practice patterns of the French chiropractic profession. We know of only one such publication, which reported that respondents to a questionnaire survey appeared to agree on a clinically responsible approach to managing cervical brachialgia [1]. We could not find studies on the same clinical problem from other parts of the world, however, preventing comparisons between geographical areas.

However, the practice pattern in relation to low back pain was studied in two previous studies conducted in Sweden [2] and Denmark [3], respectively. The Swedish chiropractors were invited with the intention of studying a group that was fairly representative of the Swedish chiropractic profession, whereas the Danish participants were selected because they were known to approve of and use maintenance care. As nearly all the Swedish chiropractors had been trained in English-speaking colleges, their clinical behaviour was expected to be influenced by the classical chiropractic tradition. In both surveys [2,3], participants were presented with information about a hypothetical patient with low back pain and several possible clinical presentations and short-term outcomes (“scenarios”) were described. Participants were asked to choose the most appropriate management strategy for each scenario. The scenarios presented in the two studies were identical.

The results of these two studies showed a fair degree of agreement on how to manage the patient with low back pain. The question then arose as to whether the French chiropractic profession would suggest the same management strategies, given the same hypothetical scenarios.

The objectives of the current study were:

1. To assess the level of consensus among French chiropractors regarding management strategies for different scenarios of low back pain

2. To assess whether the management choices of the French chiropractors appeared reasonable for different low back pain scenarios

3. To compare French management patterns in relation to low back pain with those described in a previous study of Swedish chiropractors [2].

## Method

### Study procedure

A cross-sectional survey of French chiropractors was conducted by the Institut Franco-Européen de Chiropraxie (IFEC) between 5^th^ October and 18^th^ November 2009. As there was no chiropractic legislation board in France at that time, and not all French chiropractors are members of the national chiropractic association, the exact number of practising chiropractors in France was not known. We developed a list of practitioners using the membership list of the French chiropractor association, telephone directories and the Internet. From the resulting 634 names, we extracted a random sample of 167 individuals using computer-generated random numbers.

Each potential participant received an envelope containing a letter of information, an addressed and pre-stamped envelope and a questionnaire. Participation was voluntary and anonymous. After 10 days, a second letter was sent with a request to respond to the questionnaire, if this had not already been done.

### Ethical considerations

As participation in the survey was voluntary, the survey responses were anonymous and no demographic data were collected, and there was no possibility of harm, the Ethics Committee of IFEC granted an exemption from requiring an ethics approval.

### Questionnaire

The questionnaire was the same as that used in the two previous similar studies [2,3]. A research team developed the questionnaire and pilot-tested it for user-friendliness and logic prior to the first study [2], and an accompanying interview in a second study did not reveal any misunderstandings [3]. The questionnaire was translated into French, retranslated into English, slightly amended and then finalized in the ultimate French version.

#### *The base description*

An uncomplicated case of a patient with low back pain (LBP) was used as the basis for nine hypothetical scenarios. This base description was: “A 40-year old man consults you for LBP with no additional spinal or musculoskeletal problems, and with no other health problems. His X-rays are normal for his age. There are no ‘red flags’.”

#### *Nine hypothetical scenarios*

These scenarios described various clinical presentations and short-term outcomes for the patient, varying mainly in terms of pain:

1. An acute attack of LBP of 2 days’ duration and no previous history of LBP. The pain is completely gone after two visits. The patient seems to be an uncomplicated person and able to look after himself and his back.

2. An acute attack of LBP of 2 days’ duration and no previous history of LBP. The pain is completely gone after two visits. The patient is very worried that the pain will come back again. The patient asks if he could come back regularly to make sure this will not happen.

3. An acute episode of LBP of 2 days’ duration and no previous history of LBP. The pain is about 20% better after six visits.

4. An acute episode of LBP of 1 week’s duration. The patient has had several similar attacks over the past 12 months. The pain is completely gone after two weeks of treatment.

5. An acute episode of LBP of 1 week’s duration. The patient has had several similar attacks over the past 12 months, but the pain pattern has varied over the treatment period and now, after six visits, the pain is 20% better.

6. The patient has had LBP intermittently over the past year. After the 2^nd^ visit, the pain was 50% better but today, after six visits, there has been no further change.

7. The patient has had LBP intermittently over the past year. After six visits, the pain was 80% better, but after a further two treatments in the past month, the problem has gradually become slightly worse.

8. The patient has had LBP intermittently over the past year. After the 2^nd^ visit, the pain was 20% better, but today, after six visits and over the past month, the problem has gradually become worse.

9. The patient has had LBP intermittently over the past year. After six visits, the pain is 20% better. The symptoms come and go for no apparent reason. The patient appears tired and moody.

#### *Six management strategies*

After each of the nine scenarios, six different management strategies were presented, preceded by the question: “What would you recommend?” Respondents could also suggest their own management strategy, with space available to describe it, or could tick “Don’t know”. The six management strategies (and their short titles) were:

1. I would refer the patient to another health care practitioner for a second opinion. (2^nd^ opinion)

2. I would tell the patient that the treatment is completed, but that he is welcome to make a new appointment if the problem returns. (Quick fix)

3. I would not consider the treatment to be fully completed and would try a few more treatments and perhaps change my treatment strategy, until I am sure that I cannot do any more. (Try again)

4. I would advise the patient to seek additional treatment, but would keep in touch with the patient. (External help – keep in touch)

5. I would follow this patient for a while, attempting to prolong the time period between visits until either the patient is asymptomatic or until we have found a suitable time lapse between check-ups to keep the patient symptom free. (Symptom-guided maintenance care)

6. I would recommend that the patient continue with regular visits, as long as clinical findings indicate treatment (e.g. spinal dysfunction/subluxation), even if the patient is symptom free. (Clinical findings-guided maintenance care)

7. None of the above. Please explain at the back of the page in legible handwriting. (Other)

8. Don’t know

### Expectations

In the absence of consensus documents for best clinical practice in relation to the scenarios, a research team (comprising chiropractors with research experience) had formulated some expectations on the preferred management strategy for each scenario, based on clinical experience and common sense [2]. For example, in a case of an uncomplicated problem with quick recovery, it was seen as reasonable to conclude treatment fairly quickly, whereas a longstanding and more complicated case would be expected to receive more attention and use a varied approach, perhaps including advice to obtain a second opinion.

The preferred management strategy for each scenario was as follows [2]:

Scenario 1 – Quick fix; Scenario 2 – Quick fix or Symptom-guided maintenance care; Scenario 3 – Try again; Scenario 4 - Symptom-guided maintenance care or possibly Clinical findings-guided maintenance care; Scenario 5 – Try again or External help – keep in touch; Scenario 6 – Try again or External help – keep in touch; Scenario 7 – Try again or External help – keep in touch; Scenario 8 – Second opinion; and Scenario 9 – Second opinion.

### Data analysis

The number of responses was calculated for each question. When possible, comments written under “Other” were placed in one of the pre-worded options. The option “Other” was thus only retained when this box had been ticked but no other information was provided or when such information could not be used to fit the answer into any of the other options. If the respondent selected several answers to one question, this was coded as “Other”.

Consensus was arbitrarily considered to be moderate when 50-69% of respondents agreed on the treatment option and excellent if at least 70% agreed on a treatment strategy. These cut-off points were the same as those used in the previous study on cervical brachialgia [1].

Comparisons were made with the predetermined management strategies to see whether the responses were appropriate, and with the results from the previous Swedish study [2] to determine the level of agreement between the two countries.

## Results

Of the 167 questionnaires sent out, 102 were returned of which 99 (61%) could be used. As no demographic information was available, it was not possible to compare responders and non-responders.

The most commonly selected strategies were Second opinion (n = 252 positive replies), followed by Clinical findings-guided maintenance care (n = 159) and Symptoms-guided maintenance care (n = 131). The option “Don’t know” was selected by 2-8% of respondents for each scenario.

### Was there consensus between the chiropractors on the management strategy?

As shown in Table 1, there was a noticeable pattern of common responses, but moderate consensus was noted only for scenario 2 (Clinical findings-guided maintenance care) and for scenario 9 (Second opinion). Excellent consensus was noted only for scenario 8 (Second opinion).

**Table 1:** Results from two surveys of chiropractors on choice of management strategy for low back pain

**Scenarios (Detailed description in **Method** section) and total number of respondents**	***Expected responses (Detailed description in *** Method *** section)***	***Most often selected strategy by French respondents (N = 99)***	**Agreement in French study with expected choice (Yes/No/Partial)**	***Most often selected strategy by Swedish respondents *** **[**[2]**] *(N = 59)***	**Agreement in previous Swedish study with expected choice (Yes/No/Partial)**	**Agreement between French and previous Swedish study (Yes/No/Partial)**
1 N = 96	*Quick fix*	Quick fix (41%)	Yes	*Quick fix (54%)*	Yes	Yes
2 N = 97	*Quick fix or Symptom- guided MC*	Clinical findings-guided MC (53%) (moderate consensus)	No	*Symptom-guided MC (44%)*	Yes	No
3 N = 91	*Try again*	2^nd^ opinion (21%) or try again (20%)	Partial	*Try again (42%)*	Yes	Partial
4 N = 95	*Symptom-guided MC or (possibly) Clinical findings-guided MC*	Symptom-guided MC (39%) or Clinical findings- guided MC (39%)	Yes	*Symptom-guided MC (46%)*	Yes	Yes
5 N = 95	*Try again or External help-keep in touch*	2^nd^ opinion (27%)	No	*Try again (25%) or Clinical findings- guided MC (24%)*	Yes	No
6 N = 92	*Try again or External help-keep in touch*	External help- keep in touch (27%) or try again (26%)	Yes	*Try again (37%)*	Yes	Yes
7 N = 92	*Try again or external help-keep in touch*	2^nd^ opinion (48%)	No	*Try again (32%)*	Yes	No
8 N = 92	*2*^*nd*^*opinion*	2^nd^ opinion (71%) (excellent consensus)	Yes	*2*^*nd*^*opinion (59%)*	Yes	Yes
9 N = 91	*2*^*nd*^*opinion*	2^nd^ opinion (61%) (moderate consensus)	Yes	*2*^*nd*^*opinion (59%)*	Yes	Yes

### Was the management in accordance with previously recommended strategies?

For five of the nine scenarios, the most common answers provided by the French chiropractors were in agreement with the previously recommended management strategies. For one further scenario, there was partial agreement.

Deviations from the expected answer were noted in cases where the predetermined recommended strategy was “Try again” or “External help – keep in touch”. Instead, the French chiropractors would opt for a “Second opinion”, i.e. a more defensive strategy. Another notable difference was that instead of “Quick fix” or possibly “Symptom-guided maintenance care”, about half the French chiropractors would choose “Clinical findings-guided maintenance care”, meaning that patients with benign, short-term and quickly subsiding symptoms might be subjected to long-term treatment, an approach that does not seem reasonable.

### Was the management pattern similar to that described in the Swedish survey?

In both the French and Swedish studies there was a noticeable pattern of common responses, but moderate or excellent consensus in only three cases. In the Swedish study, however, the most common answer was always in agreement with the recommended expected strategy, whereas this was the case in five (plus one partial agreement) of the nine scenarios in the French study. Consensus between the French and Swedish responses on the most appropriate management was seen in five of the scenarios (plus one partial agreement) and these were all in agreement with the expected answer.

## Discussion

This study investigated the choices French chiropractors would make in relation to nine specific scenarios for managing low back pain. Our findings indicate that the chiropractors would often either send patient for a second opinion or retain them in a clinical findings-guided maintenance care programme. Although the chiropractors agreed reasonably well on the proposed strategies, this agreement reached moderate consensus for only two of the nine scenarios, and excellent consensus for one scenario. The choices were considered reasonable in five of the nine strategies (on the basis of previously determined “best practice”). The number of moderate and excellent agreements was similar to the results in the Swedish study, but the French responses deviated more from the expected responses than in the Swedish study. The French and Swedish chiropractors agreed with each other on the most common answer in five of the nine scenarios and all these were in line with “best practice”.

Interestingly, one of the items on which there was high consensus in the French study was out of line with the expected answer. The scenario was that of a patient with an acute attack of low back pain of two days’ duration and no previous history of low back pain. The pain was described as completely gone after two visits, but the patient was worried that the pain would return, asking if he could return for regular treatment to ensure that this would not happen. The expected “best practice” answer was either to finish the treatment, explaining that the patient would be welcome back again if needed (Quick Fix) or, in view of his anxiety, to invite the patient for a couple of follow-ups, basing the treatment approach on the patient’s degree of pain (Symptom-guided maintenance care). Instead, about half of the French chiropractors would choose to ask this patient to return for maintenance care, basing the treatment on clinical findings. The corresponding result was 30% in the Swedish study. This approach of clinical finding-guided maintenance care is unlikely to be the most appropriate management for this patient, taking into account the lack of objective tests that can be performed on “silent” backs. Only 28% of the French chiropractors would have treated this patient with one of the “best practice” options.

The other answers that deviated from the expected responses were all in favour of referring for a second opinion rather than attempting further treatment or advising supplementary external treatment. In fact, similarly to the previous French study on brachialgia [1], contact with other health professionals was often suggested, and “External help” or “Second opinion” were selected in six of the nine scenarios. The Swedish chiropractors preferred these two options only twice. This may be a peculiarity of the French situation. The long history of having been an illegal profession with limited collaboration and feedback from other health care practitioners may have resulted in a more defensive practice style, with less self-confidence.

In both the French and Swedish studies, there was a noticeable pattern of common responses but moderate or excellent consensus was noted in only three cases. In the Swedish study, the most common answers were always in agreement with the expected response, whereas this was the case in five of the nine scenarios in the French study. The Swedish study sample may thus have included more “mainstream” chiropractors. It is not known whether this is a reflection of the entire Swedish profession or if it is due to selection bias. Our results suggest, however, that sometimes similar patients presenting in different clinics could experience quite different management approaches both within France and between countries. These practice variations need to be addressed at undergraduate and postgraduate levels of chiropractic training.

### Methodological considerations

Although invitation to this study was based on random selection, it is not known whether the sampling frame consisted of all chiropractors in France. We consider the response rates to be quite low in both our study and the Swedish study (about 60%), and it is possible that the non-responders would have different opinions about the most appropriate treatment for the patient scenarios described. We collected the data on an anonymous basis to boost the response rate, but demographic and professional information on the study sample would have been useful to clarify potential differences between responders and non-responders.

The questionnaire was translated (through forward and back translation) and accommodated to the French-speaking culture, and although we aimed at an equivalent translation, there may have been differences in interpretation between the French and Swedish versions. The questionnaire seemed to have been well received by the respondents, as there were very few comments and questions on the questionnaire text and no apparent treatment options that were lacking.

## Conclusion

This cross-sectional postal survey found reasonable consensus among French chiropractors in their choice of management strategy for low back pain. In general, their choice of treatment strategy was also in line with predetermined “best practice”. It would be useful, however, to initiate discussion on the use of maintenance care, in particular for patients with a benign history of low back pain and no symptoms.

The greater consensus on management strategies among Swedish chiropractors suggests that cultural and/or educational differences influence the conceptual framework within which chiropractors practice. There appears to be no generally accepted chiropractic management strategy for low back pain.

## Competing interests

The authors declare that they have no competing interests.

## Authors’ contributions

This article is based on a research project conducted as a part requirement for the chiropractic degree at the Institut Franco-Européen de Chiropraxie by RA, LD, AG and TV. Supervision was provided by MD and CL. Data were re-analyzed and the article version of the report was drafted by CL. All authors read and approved the final version.
